# Assessment of Discordance Between Physicians and Family Members Regarding Prognosis in Patients With Severe Acute Brain Injury

**DOI:** 10.1001/jamanetworkopen.2021.28991

**Published:** 2021-10-21

**Authors:** Whitney A. Kiker, Rachel Rutz Voumard, Leah I. B. Andrews, Robert G. Holloway, Lyndia C. Brumback, Ruth A. Engelberg, J. Randall Curtis, Claire J. Creutzfeldt

**Affiliations:** 1 Division of Pulmonary, Critical Care and Sleep Medicine, University of Washington, Seattle; 2 Cambia Palliative Care Center of Excellence, University of Washington, Seattle; 3 Harborview Medical Center, Department of Neurology, University of Washington, Seattle; 4 Palliative and Supportive Care Service, Lausanne University Hospital, University of Lausanne, Lausanne, Switzerland; 5 Department of Biostatistics, University of Washington School of Public Health, Seattle; 6 Department of Neurology, University of Rochester Medical Center, Rochester, New York

## Abstract

**Importance:**

Shared decision-making requires key stakeholders to align in perceptions of prognosis and likely treatment outcomes.

**Objective:**

For patients with severe acute brain injury, the objective of this study was to better understand prognosis discordance between physicians and families by determining prevalence and associated factors.

**Design, Setting, and Participants:**

This mixed-methods cross-sectional study analyzed a cohort collected from January 4, 2018, to July 22, 2020. This study was conducted in the medical and cardiac intensive care units of a single neuroscience center. Participants included families, physicians, and nurses of patients admitted with severe acute brain injury.

**Exposures:**

Severe acute brain injury was defined as stroke, traumatic brain injury, or hypoxic ischemic encephalopathy with a Glasgow Coma Scale score less than or equal to 12 points after hospital day 2.

**Main Outcomes and Measures:**

Prognosis discordance was defined as a 20% or greater difference between family and physician prognosis predictions; misunderstanding was defined as a 20% or greater difference between physician prediction and the family’s estimate of physician prediction; and optimistic belief difference was defined as any difference (>0%) between family prediction and their estimate of physician prediction. Logistic regression was used to identify associations with discordance. Optimistic belief differences were analyzed as a subgroup of prognosis discordance.

**Results:**

Among 222 enrolled patients, prognostic predictions were available for 193 patients (mean [SD] age, 57 [19] years; 106 men [55%]). Prognosis discordance occurred for 118 patients (61%) and was significantly more common among families who identified with minoritized racial groups compared with White families (odds ratio [OR], 3.14; CI, 1.40-7.07, *P* = .006); among siblings (OR, 4.93; 95% CI, 1.35-17.93, *P* = .02) and adult children (OR, 2.43; 95% CI, 1.10-5.37; *P* = .03) compared with spouses; and when nurses perceived family understanding as poor compared with good (OR, 3.73; 95% CI, 1.88-7.40; *P* < .001). Misunderstanding was present for 80 of 173 patients (46%) evaluated for this type of prognosis discordance, and optimistic belief difference was present for 94 of 173 patients (54%). In qualitative analysis, faith and uncertainty emerged as themes underlying belief differences. Nurse perception of poor family understanding was significantly associated with misunderstanding (OR, 2.06; 95% CI, 1.07-3.94; *P* = .03), and physician perception with optimistic belief differences (OR, 2.32; 95% CI, 1.10-4.88; *P* = .03).

**Conclusions and Relevance:**

Results of this cross-sectional study suggest that for patients with severe acute brain injury, prognosis discordance between physicians and families was common. Efforts to improve communication and decision-making should aim to reduce this discordance and find ways to target both misunderstanding and optimistic belief differences.

## Key Points

**Question:**

For patients with severe acute brain injury, how prevalent is prognosis discordance between physicians and families, and what factors are associated with it?

**Findings:**

In this cross-sectional study of 193 patients with severe acute brain injury, prognosis discordance occurred for 61% of patients, with both misunderstanding and belief differences present; prognosis discordance was more likely for family members who were part of minoritized racial groups. Nurses seemed to accurately predict poor family understanding, whereas physicians perceived belief differences as poor family understanding.

**Meaning:**

The results of this cross-sectional study suggest that prognosis discordance, which may hamper the shared decision-making process, is common between families and physicians for patients with severe acute brain injury.

## Teaser

This cross-sectional study uses data from a single neuroscience center to assess for the presence of discordance between physicians and family members regarding prognosis in patients with severe acute brain injury.

## Introduction

Effective shared decision-making ideally requires clinicians, patients, and families to have a mutual understanding of patient and family values, and of the patient’s most likely prognosis and outcomes of treatment options.^1,2,3^ Substantial differences between prognostic perceptions of these stakeholders may compromise the patient’s or family’s ability to make an informed decision that is value-concordant to the individual patient.^4^ Although the role of effective communication is evident in creating a shared understanding of prognosis, discussions about prognosis are challenging for everyone involved.^5,6^ Families of critically ill patients do not solely rely on information provided by physicians but also incorporate their own beliefs about the patient to draw conclusions about prognoses.^7,8^ Prognostic estimates of physicians and families differ frequently, and misunderstanding between parties is common.^9,10^

Prognostic uncertainty is particularly high after severe acute brain injury (SABI), a group of devastating neurologic conditions including stroke, traumatic brain injury, and hypoxic ischemic encephalopathy after cardiac arrest.^11,12,13^ Patients with SABI often require life-or-death treatment decisions early on during their hospital stay but are unable to participate in the decision-making process themselves. Family members are tasked with participating in highly consequential treatment decisions by integrating medical and prognostic information with their loved one’s presumed goals of care.^14^

Prognostic discordance between families and physicians specific to SABI is poorly understood. Given that SABI threatens not only a patient’s life but also their personhood and quality of life, prognosis as it pertains to functional recovery may be more relevant than survival alone.^15^ The objective of this study was to determine the prevalence and etiology of prognostic discordance between physicians and family members of patients with SABI and to explore the association between discordance and patient and family characteristics.

## Methods

### Design

Participants were part of a prospective cross-sectional study conducted from January 4, 2018, to July 22, 2020, in the neurosciences and medical and cardiac intensive care units (ICUs) of a comprehensive stroke and level I trauma center in the US Pacific Northwest. Data were collected through in-person surveys of physicians, nurses, and families during the acute hospitalization as well as through review of the electronic health record. Study data were managed using REDCap electronic data capture tools^16^ hosted at the Institute of Translational Health Sciences. This study was approved by the institutional review board of the University of Washington and followed the Strengthening the Reporting of Observational Studies in Epidemiology (STROBE) reporting guideline.

### Participants and Enrollment

Patients were screened after hospital day 2 and deemed eligible if they were admitted with a diagnosis of SABI, defined as stroke (ischemic stroke, intraparenchymal hemorrhage or subarachnoid hemorrhage), traumatic brain injury, or hypoxic ischemic encephalopathy after cardiac arrest. Eligibility criteria also included a Glasgow Coma Scale score of 12 points or less after hospital day 2 and having an English-speaking family member available in the hospital or by telephone. Family members of every eligible patient were invited to participate once the attending physician of the primary medical team granted permission. Because patients were unable to consent for themselves, their durable power of attorney or legal next of kin was asked for informed consent for the patient’s participation and access to their electronic health record. Cohort size for this exploratory study was determined by patient eligibility and family member participation during the enrollment period for the larger study. If multiple family members were present, the person who took chief responsibility for medical decision-making completed the survey. The attending physician and bedside nurse caring for the patient on the day of enrollment were also approached for participation.

### Data Collection

Data were collected from families, physicians, and nurses via surveys, whereas patient data were collected via review of the electronic health record. Family members were invited between hospital days 2 and 7 to complete a survey with 3 questions regarding the patient’s prognosis (Table 1). First, we asked the family member to predict the likelihood of the patient recovering to independence. The response option was a visual analog scale from 0% to 100%. Second, we asked the family member to estimate what the attending physician would predict if asked the same question. If the family member’s prediction was different from what they estimated the physician would predict, we asked the family member what might explain this difference. This third question was open-ended, and the response was documented word for word. Additional questions for the families included questions about their own demographic information and about how much they trusted the information received on a scale from 0 (not at all) to 10 (completely).

**Table 1.** Survey Questions

We asked: “Looking at 6 mo or so from now…”	Response option
Family members	
“What do you think your loved one’s chance is of recovering to the point of independence (able to interact, feed, and bathe without anyone else’s assistance) or better?”	Visual analog scale from 0% to 100%
“If you had to guess, what do you think the doctor thinks your loved one’s chance is of recovering to independence?”	Visual analog scale from 0% to 100%
“What explains the difference between what you think and what your doctor thinks is the chance of recovering to independence?”	Open-ended, documented word for word
“How much do you trust the medical information you have received?”	Visual analog scale from 0 to 10
Physicians	
“What do you think the patient’s chance is of recovering to the point of independence (able to interact, feed, and bathe without anyone else’s assistance) or better?”	Visual analog scale from 0% to 100%
“How would you rate the quality of the family’s understanding of prognosis?”	Likert scale: poor, fair, good, very good, or excellent
Nurses	
“How would you rate the quality of the family’s understanding of prognosis?”	Likert scale: poor, fair, good, very good, or excellent

That same day, we asked the attending physician to predict the likelihood of the patient recovering to independence, using the same response options as for the family. Additionally, we asked the physician and bedside nurse to rate the quality of the family’s understanding of prognosis on a 5-point Likert scale (“poor,” “fair,” “good,” “very good,” or “excellent”). Families and nurses were surveyed in person or by telephone by research staff, and attending physicians were surveyed either in person or by email. Physicians, families, and nurses were blinded to the responses of the others. All surveys were completed on the day of enrollment, as many consequential decisions are made for patients with SABI during the first week of hospitalization.^17^ Because discussions about prognosis are often held informally through bedside updates and formal family meetings were inconsistently documented in the electronic health record, we did not capture whether or to what extent prognosis discussions had occurred prior to the survey.

### Outcomes

#### Prognosis Discordance and Misunderstanding

Overall prognosis discordance was defined as a difference of at least 20% between family prognosis prediction and physician prognosis prediction. We chose an absolute difference of 20% or greater because it (1) allowed for subtle variations in prognosis predictions to be treated as concordant and (2) was used in a previous study examining prognostic discordance between physicians and families regarding survival in the general ICU.^7^ We also defined optimistic prognosis discordance as prognosis discordance in which the family member was more optimistic than the physician. This distinction was made based on literature suggesting that optimism among family members relative to physicians is substantially more common than pessimism, and concern that factors associated with family optimism would differ from factors associated with family pessimism.^7^ Misunderstanding of prognosis was defined as a difference of at least 20% between the family’s estimate of the physician’s prediction and the physician’s actual prediction.

#### Optimistic Belief Difference

Optimistic belief difference was defined as a family member’s own prediction that was greater than what they estimated the physician would predict. We chose greater than 0% as the criterion for optimistic belief difference because this comparison was between 2 responses given by the same person. Therefore, any difference in the responses was deliberate. In our analysis, we considered only the subset of participants with optimistic belief difference relative to the subset with no belief difference, excluding those with pessimistic belief difference.

### Variables of Interest

Our analyses explored associations between the outcomes and 9 variables: patient age and disease category; family member sex, race, ethnicity, and relationship to patient; nurse rating of family understanding; physician rating of family understanding; and family level of trust in information received. Variables were selected if they might (1) be associated with the patient’s prognosis (patient age and disease category), (2) contribute to the family’s perception of prognosis (family member sex, race, ethnicity, and relationship to patient), or (3) be associated with discordance (physician and nurse perception of family understanding and family level of trust). Family member race was dichotomized into “White” and “minoritized racial groups,” with the latter category including those who identified as Asian, Black or African American, Native American, Pacific Islander, or another unspecified race. We dichotomized physician and nurse rating variables into fair or worse vs good or better; we assumed nonresponse indicated a lack of strong positive feelings and included nonresponse in the “fair or worse” category. The family trust variable was dichotomized into 10 vs 0 to 9 (<10); we assumed nonresponse indicated a lack of strong positive feelings and included nonresponse in the “less than 10”category.

### Statistical Analysis

We fit several logistic regression models to evaluate the association between the measures of discordance and the 9 variables of interest. Model 1 was an unadjusted logistic regression model for each of those 9 variables and each outcome; results were similar to those of adjusted models (eTable in the Supplement). Model 2 included patient age and disease category and family sex, race, and ethnicity together for each of the outcomes, in order to adjust for potential confounding. Model 3 included all Model 2 variables and 1 additional variable of interest (relationship, trust in the information received, physician perception, or nurse perception of family member understanding). Model 3 variables were also considered potential confounders and were added separately owing to potential collinearity. Patients were included in the evaluation of each outcome only if complete data were available (eFigure in the Supplement). A *P* value of .05 was used to indicate statistical significance. A *t* test with unequal variance was used for continuous variables, and a χ^2^ test for independence was used for categorical variables. Statistical analyses were conducted using R, version 3.6.2 (R Foundation) and Stata, version 14.2 (StataCorp).

#### Qualitative Analysis

Family members’ responses to open-ended questions about optimistic belief differences were analyzed using content analysis.^18,19^ Three investigators (W.A.K., R.R.V., and C.J.C.) independently reviewed responses, and each developed a coding scheme that grouped response concepts into categories. Following initial determination of codes, investigators collaborated to determine a final coding framework and then coded responses based on this established framework. Trustworthiness was established by reviewing codes with an additional investigator (R.A.E.).

## Results

### Enrollment

Among the 222 enrolled patients, prognostic predictions were available for 193 patients (mean [SD] age, 57 [19] years; 106 men [55%]; 8 American Indian or Alaskan Native [4%]; 18 Asian [9%]; 17 Black [9%]; 2 Hawaiian or Pacific Islander [1%]; 148White [77%]). A total of 45 patients (23%) were in the minoritized racial group category. The prognosis question was completed by both families and physicians for 193 of 222 patients (87%), who had similar demographic characteristics (Table 2). Twenty family members (10%) did not provide their estimate of physician prediction, leaving 173 patients for whom misunderstanding and optimistic beliefs could be analyzed (eFigure in the Supplement).

**Table 2.** Baseline Characteristics for Patients and Family Members

Characteristic	Participants, No. (%)	
	Patient (n = 193)	Family (n = 193)
Age, mean (SD), y	57 (19)	51 (17)
Sex		
Women	87 (45)	126 (65)
Men	106 (55)	67 (35)
Race		
American Indian/Alaskan Native	8 (4.1)	4 (2.1)
Asian	18 (9.3)	15 (7.8)
Black	17 (8.8)	18 (9.3)
Hawaiian/Pacific Islander	2 (1.0)	8 (4.1)
White	148 (76.7)	143 (74.1)
Other race	NA	5 (2.6)
Ethnicity		
Hispanic	14 (7)	20 (10)
Admission diagnosis		
Ischemic stroke	37 (19)	NA
Intraparenchymal hemorrhage	35 (18)	
Subarachnoid hemorrhage	46 (24)	
Traumatic brain injury	56 (29)	
Cardiac arrest	19 (10)	
Glasgow Coma Scale score, mean (SD), points	7 (3)	
Relationship		
Spouse	NA	57 (30)
Parent		29 (15)
Sibling		21 (11)
Child		68 (35)
Other^a^		18 (9)
Physician perception of fair or worse family understanding		71 (37)
Nurse perception of fair or worse family understanding		82 (42)
Family trust in information received <10^b^		72 (37)

Abbreviation: NA, not applicable.

^a^ Other relationship includes a domestic partner, niece or nephew, aunt or uncle, or cousin.

^b^ Refers to a score on the ordinal scale reflecting how much trust a family has in the information received.

### Discordance Measures

**Figure 1.**
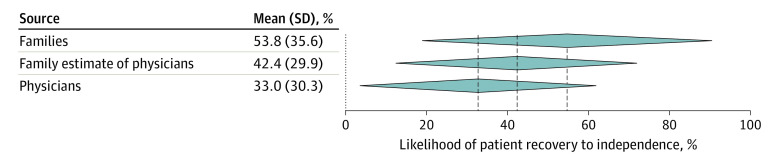
Mean Prediction of Prognosis by Group, With SD

Overall prognosis discordance between physicians and families occurred for 118 of 193 patients (61%). Among those, 99 (84%) represented optimistic discordance, and 19 (16%) represented pessimistic discordance. Misunderstanding occurred for 80 of 173 patients (46%). Optimistic belief difference occurred for 94 of 173 patients (54%), pessimistic belief difference for 15 of 173 (9%), and no belief difference for 64 of 173 patients (37%). Among this group of 173 patients, the mean (SD) value for family prediction for a patient’s likelihood of recovering to independence was 54% (36%); for family estimate of physician prediction, 42% (30%); and for physician prediction, 33% (30%) (Figure 1).

### Factors Associated With Outcomes

#### Prognosis Discordance

**Figure 2.**
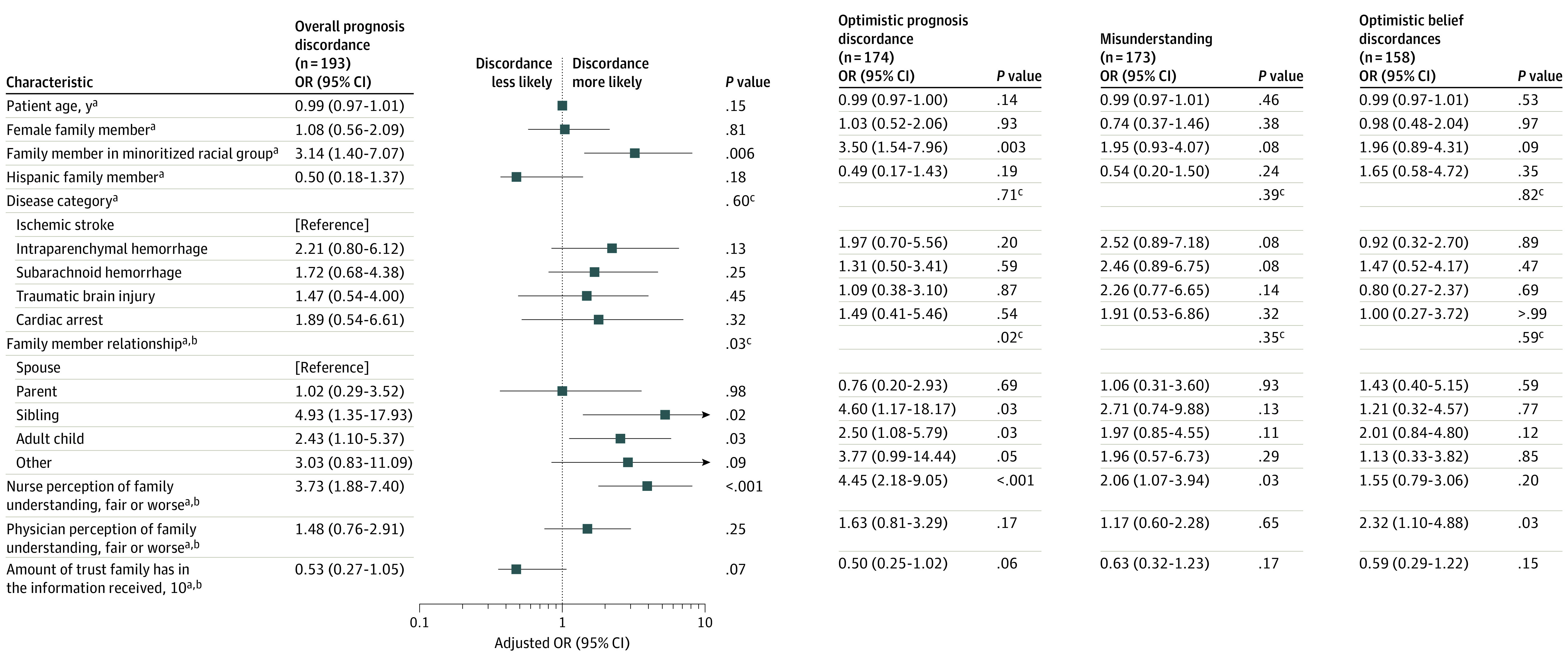
Adjusted Associations for Different Types of Discordance Missing values for nurse perception of family understanding (n = 15) and for physician perception of family understanding (n = 5) were assumed to represent a lack of strong positive feelings and were therefore included in the “fair or worse” category. Missing values for the family trust variable (n = 14) were assumed to represent a lack of strong positive feelings and therefore included in the <10 category. OR indicates odds ratio. The minoritized racial groups category includes participants who identified as American Indian/Alaskan Native, Asian, Black or African American, Hawaiian/Pacific Islander, or another unspecified race. ^a^All models adjusted for patient age, disease category, family member sex, family member race, and family member ethnicity. ^b^These models also adjusted for the specific potential predictor variable. ^c^Overall *P* value for group.

Among the entire sample of 193 patients, the odds of overall prognosis discordance were 3.14 times (95% CI, 1.40-7.07; *P* = .006) higher for family members in minoritized racial groups compared with White family members, after adjusting for patient age, disease category, family sex, and family ethnicity (Figure 2). The odds of optimistic prognosis discordance were also higher for minoritized racial groups compared with White family members (OR, 3.50; 95% CI, 1.54-7.96; *P* = .003) among the 174 patients with optimistic or no prognosis discordance. With regard to family relationship, the odds of prognostic discordance were 2.43 times higher for adult children (95% CI, 1.10-5.37; *P* = .03) and 4.93 times higher for siblings (95% CI, 1.35-17.93; *P* = .02) compared with spouses. Nurse perception of fair or worse family member understanding was independently associated with overall prognosis discordance (OR, 3.73 compared with good understanding or better; 95% CI, 1.88-7.40; *P* < .001) and optimistic prognosis discordance (OR, 4.45 compared with good understanding or better; 95% CI, 2.18-9.05; *P* < .001).

#### Misunderstanding

Among the 173 patients for whom families provided their estimate of physician prediction, nurse perception of fair or worse family understanding was associated with family misunderstanding (OR, 2.06 compared with good understanding or better; 95% CI, 1.07-3.94; *P* = .03). Physician perception of fair or worse family understanding was not significantly associated with family misunderstanding (OR, 1.17 compared with good understanding or better; 95% CI, 0.60-2.28; *P* = .65).

#### Optimistic Belief Difference

Among the 158 patients for whom optimistic belief difference or no belief difference was present, physician perception of fair or worse family understanding was associated with optimistic belief difference (OR, 2.32 compared with good or better understanding; 95% CI, 1.10-4.88; *P* = .03). Nurse perception of family understanding was not associated with optimistic belief difference (OR, 1.55 comparing perceived fair or worse with perceived good or better understanding; 95% CI, 0.79-3.06; *P* = .20).

### Family’s Explanation of Optimistic Belief Difference

For the 94 patients with optimistic belief difference, qualitative analysis of the open-ended comments revealed 2 key themes. First, families described relying on various sources of faith that the patient would have a better outcome than might be expected. These sources included faith in God, in the individual strengths of their loved one, and in the power of optimism to effect a favorable outcome. Second, families identified prognostic uncertainty as a justification for their optimistic beliefs. Uncertainty encompassed a general understanding that the patient’s chance of recovery was uncertain, a feeling that their own lack of medical knowledge led to uncertainty, and a sense that the physician’s prognostication was cautious owing to uncertainty (Table 3).

**Table 3.** Qualitative Analysis of Optimistic Belief Differences

Reason	Example
Faith	
Faith in God	“I am looking at it from the spiritual perspective. We know that God is going to heal her.”
	“He’s a doctor and sees the science, but [...] God isn’t finished with him yet.”
Optimism and hope	“I need to be more optimistic for her […] Doctors don’t see the family support—her family being there for her is going to help her.”
	“I’m hoping; putting faith in what could be and staying positive.”
Faith in patient strength	“We want to stay hopeful. We know his perseverance and we also have a strong faith.”
	“We’re hopeful and know she is strong and will give all she can to get better.”
Uncertainty	
Family uncertainty	“So much is unknown.”
	“It’s hard to know what is going to happen because it is so early.”
Family uncertainty of medical facts	“I haven’t been through this before, the doctors have done this many times.”
	“… they are the ones with all the data.”
Physician uncertainty	“The doctors are walking a fine line between being honest […] and trying to be optimistic, but they’re not very optimistic.”
	“The doctors have to be like that, because they don’t know. We, as the family, have to keep hope or he will lose hope.”

## Discussion

In this cross-sectional study of patients with SABI, we found a high prevalence of physician-family discordance regarding a patient’s likelihood of recovering to independence. The prevalence of overall prognosis discordance in our study (61%) was slightly higher than that found in a study of survival prognosis discordance in a general ICU population (53%).^7^ Although this may reflect a higher prevalence of uncertainty in the SABI population relative to the general ICU population, direct comparisons of the 2 studies are difficult. Without information about which group of participants was more accurate in their prognosis, the high prevalence of prognosis discordance after SABI represents opportunities to improve communication and shared decision-making by better aligning physician and family prognostic perceptions.

Our findings suggest that both misunderstanding and optimistic belief difference play a role in prognosis discordance. Misunderstanding may indicate ineffective prognosis communication for a variety of reasons. For example, physicians often fail to ensure family understanding, they rarely use numbers to convey prognosis,^20^ and families may interpret indirect comments as optimism.^21^ Mitigating such pitfalls might reduce prognosis discordance. Optimistic belief difference, on the other hand, represents an intentional decision by a family member to believe a different prognosis than what the physician may have communicated. Optimistic belief difference may reflect a feeling of hope, which has been identified as an important coping mechanism, especially in the face of uncertainty.^14,22,23^ Our qualitative analysis suggests that families choose hope for the benefit of both the patient and themselves: “we have to keep hope, or [the patient] will lose hope.” Therefore, it may not be feasible or helpful to attempt to eliminate this component of discordance. Although physicians may perceive optimistic beliefs as a false hope that hinders the decision-making process, for families it may be a crucial support in a desperate time. Distinguishing between these 2 types of prognosis discordance may aid the development of future interventions to improve shared decision-making by addressing misunderstanding and optimistic beliefs differently.

Physician rating of poor family understanding of prognosis was associated with optimistic belief difference in the subgroup analysis but not with the other discordance measures. Nurse rating of poor understanding was associated with overall prognosis discordance and with misunderstanding but not with optimistic belief difference. Physicians may misinterpret optimism or hope as a lack of understanding, whereas nurses may more accurately identify misunderstanding. This finding is important given that nurses are not always part of decision-making but do spend more time at the bedside than physicians^24^ and are frequently engaged in conversations about prognosis with family members.^25,26^ Prognostic predictions by nurses as well as interventions to encourage nurses’ involvement in the shared decision-making process should be further investigated.

Families who identified as minoritized racial groups were significantly more likely to experience overall prognosis discordance. Previous studies have found that Black patients are more likely to experience poor clinician communication compared with White patients, particularly when clinicians are of a different race,^27,28^ and that being a member of a minoritized racial group is associated with lower palliative care use after SABI.^29,30^ The quality of physician communication for individuals of minoritized racial groups or non–race-concordant families may contribute to misunderstanding. Patients and families of minoritized racial groups are also more likely to experience lack of trust in and dissatisfaction with the health care system,^31,32,33^ which could facilitate increased optimistic belief difference. Demonstrating trustworthiness and building trust with families of minoritized racial groups, which includes increasing the proportion of physicians of minoritized racial groups (or with race concordance), may be an avenue for mitigating prognostic discordance.

### Strengths and Limitations

Strengths of this study include the survey methodology, in which participants were asked about real patients in real time. The mixed quantitative and qualitative questions allowed us to measure prevalence of discordance while also exploring family member views of contributing factors. The near simultaneous timing of the surveys of families and physicians ensured that prognosis had not changed and facilitated blinding each party to the other’s response.

This study has important limitations. First, the study was conducted at a single hospital in the US Pacific Northwest, which may limit the generalizability of the results. The hospital is the only comprehensive stroke and level I trauma center in a 5-state region, perhaps minimizing those limits. Second, the collection of data early during the ICU stay may have missed improved concordance later. We deliberately chose this early time frame (1) in order to ensure a broad range of patients and conditions and (2) because many pivotal treatment decisions are made during this period.^17^ Third, we asked only the attending physician and 1 family member for their prognosis predictions, and it is possible that multiple physicians, including residents and other attending physicians, as well as other family members were involved in prognosis discussions. Interphysician variability in prognostication could have accounted for some differences in prognosis perceptions, although that should be mitigated by the selection of a 20% window for discordance. Fourth, we did not track response rates by individual clinicians, which risked clustering of predictions. However, the high number of physicians who responded makes this less likely. Fifth, we did not include family member education or health literacy as a variable, which could have been a confounder. Finally, optimistic prognosis discordance and optimistic belief difference were subgroups of the larger cohort defined by the outcome, and therefore results related to these outcomes could have been biased. However, they were included to allow for a more focused evaluation of optimistic discordance vs concordance.

## Conclusions

In this cross-sectional study, for patients with SABI, discordance was common between physicians and family members regarding their likelihood of recovering to independence, which suggests opportunities to improve communication and shared decision-making. Further research is needed to measure the relative accuracy of prognostic predictions for families and physicians after SABI and to examine whether reducing prognosis discordance can improve decision-making and long-term outcomes for patients and family members.

## Notes

Supplement. **eFigure.** Enrollment and Participation of Families, Physicians, and Nurses **eTable.** Unadjusted Associations for Different Types of Discordance
