# Supplementary material for: Assessment of Discordance Between Physicians and Family Members Regarding Prognosis in Patients With Severe Acute Brain Injury
Source: JAMA Netw Open. 2021 Oct 21;4(10):e2128991. doi: 10.1001/jamanetworkopen.2021.28991 (PMC8531991; doi:10.1001/jamanetworkopen.2021.28991)
Supplement: Supplement. — eFigure. Enrollment and Participation of Families, Physicians, and Nurses eTable. Unadjusted Associations for Different Types of Discordance [file jamanetwopen-e2128991-s001.pdf]

Supplementary Online Content

Kiker WA, Rutz Voumard R, Andrews LIB, et al. Assessment of discordance between physicians and family members regarding prognosis in patients with severe acute brain injury. *JAMA Netw Open*. 2021;4(10):e2128991. doi:10.1001/jamanetworkopen.2021.28991

eFigure. Enrollment and Participation of Families, Physicians, and Nurses

eTable. Unadjusted Associations for Different Types of Discordance

This supplementary material has been provided by the authors to give readers additional information about their work.

© 2021 Kiker WA et al. *JAMA Network Open.*

eFigure. Enrollment and Participation of Families, Physicians, and Nurses

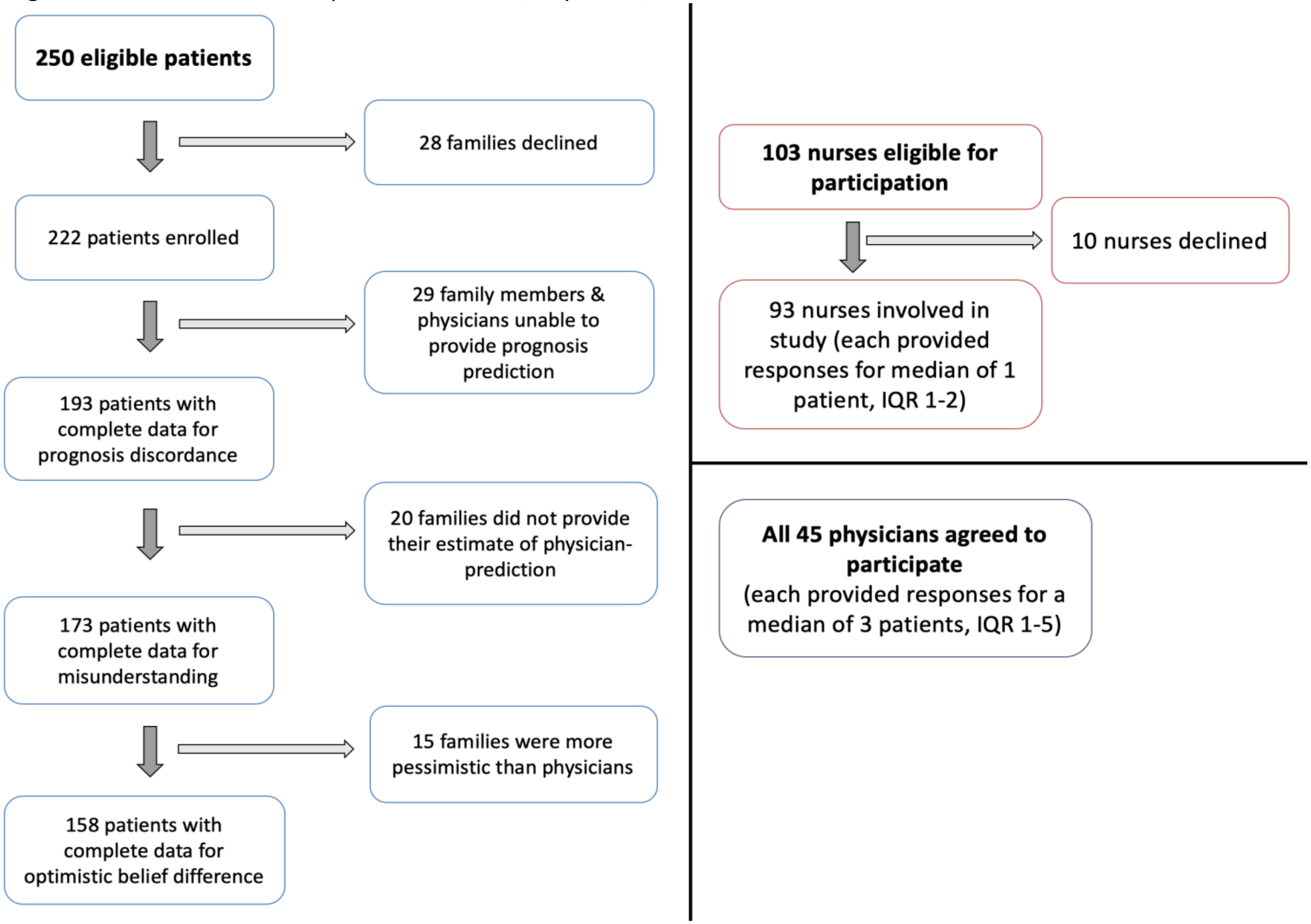

Patients were only included in the evaluation of each outcome if complete data were available. For evaluation of misunderstanding, family-physician pairs were only included when physicians provided their prediction and families provided an estimate for physician-prediction (n=173). For evaluation of optimistic belief difference (vs no difference), we only included pairs where families provided both their own prognosis prediction and an estimate for physician-prediction, and we excluded those with pessimistic belief difference, leaving 158 patients.

© 2021 Kiker WA et al. *JAMA Network Open.*

eTable. Unadjusted Associations for Different Types of Discordance

	OVERALL PROGNOSIS DISCORDANCE (n=193)		OPTIMISTIC PROGNOSIS DISCORDANCE (n=174)		MISUNDERSTANDING (n=173)		OPTIMISTIC BELIEF DIFFERENCES (n=158)	
**Characteristic**	**OR (95% CI)**	**P Value**	**OR (95% CI)**	**P Value**	**OR (95% CI)**	**P Value**	**OR (95% CI)**	**P Value**
**Patient Age, years**	**0.98 (0.97,1)**	**0.03**	**0.98 (0.97,1)**	**0.04**	0.99 (0.97,1)	0.11	0.99 (0.98,1.01)	0.46
**Female family member**	1.21 (0.66,2.21)	0.54	1.14 (0.61,2.13)	0.68	0.84 (0.45,1.59)	0.59	1.04 (0.53,2.05)	0.91
**BIPOC family member**	**3.33 (1.55,7.18)**	**<0.01**	**3.88 (1.78,8.47)**	**<0.01**	1.86 (0.94,3.66)	0.07	**2.12 (1.01,4.46)**	**0.05**
**Hispanic family member**	0.6 (0.24,1.52)	0.28	0.57 (0.21,1.53)	0.26	0.59 (0.22,1.56)	0.29	1.55 (0.56,4.32)	0.40
**Disease Category**		0.32 [a]		0.39 [a]		0.28 [a]		0.68 [a]
Ischemic Stroke	(Reference)		(Reference)		(Reference)		(Reference)	
Intra-parenchymal hemorrhage	2.3 (0.88,6.03)	0.09	2.11 (0.8,5.56)	0.13	2.44 (0.89,6.7)	0.08	1.02 (0.37,2.86)	0.97
Subarachnoid hemorrhage	1.8 (0.75,4.34)	0.19	1.43 (0.58,3.51)	0.44	2.56 (0.96,6.8)	0.06	1.51 (0.55,4.16)	0.43
Traumatic brain injury	1.63 (0.71,3.77)	0.25	1.2 (0.51,2.84)	0.68	2.13 (0.85,5.35)	0.11	0.89 (0.36,2.2)	0.80
Cardiac arrest	2.29 (0.72,7.31)	0.16	1.94 (0.59,6.33)	0.28	2.04 (0.61,6.84)	0.25	1.08 (0.31,3.8)	0.90
**Family Member Relationship**		**0.04 [a]**		**0.03 [a]**		0.40 [a]		0.62 [a]
Spouse/Partner	(Reference)		(Reference)		(Reference)		(Reference)	
Mother/Father	2.27 (0.9,5.72)	0.08	1.97 (0.74,5.25)	0.17	1.61 (0.63,4.08)	0.32	1.78 (0.67,4.76)	0.25
Sister/Brother	**5.07 (1.51,16.95)**	**<0.01**	**4.58 (1.32,15.93)**	**0.02**	2.58 (0.79,8.45)	0.12	1.4 (0.41,4.71)	0.59
Son/Daughter	**2.05 (1,4.2)**	**0.05**	2.09 (0.99,4.4)	0.05	1.57 (0.73,3.36)	0.25	2.1 (0.93,4.72)	0.08
Other	3.1 (0.98,9.85)	0.06	**3.66 (1.14,11.77)**	**0.03**	1.94 (0.64,5.91)	0.25	1.5 (0.48,4.66)	0.49
**Nurse perception of family understanding, fair or worse**	**3.75 (1.98,7.13)**	**<0.01**	**4.48 (2.31,8.7)**	**< .01**	**2.1 (1.13,3.89)**	**0.02**	1.68 (0.87,3.24)	0.12
**Physician perception of family understanding, fair or worse**	1.61 (0.87,2.96)	0.13	1.74 (0.92,3.26)	0.09	1.27 (0.68,2.36)	0.46	**2.53 (1.25,5.13)**	**0.01**
**Amount of Trust Family has in the Information Received**	0.69 (0.37,1.26)	0.23	0.64 (0.34,1.2)	0.16	0.75 (0.4,1.39)	0.36	0.61 (0.31,1.2)	0.15

BIPOC indicates black, indigenous, and people of color; [a] Overall p value for group

© 2021 Kiker WA et al. *JAMA Network Open.*
